# Modeling Apple Surface Temperature Dynamics Based on Weather Data

**DOI:** 10.3390/s141120217

**Published:** 2014-10-27

**Authors:** Lei Li, Troy Peters, Qin Zhang, Jingjin Zhang, Danfeng Huang

**Affiliations:** 1 School of Mechanical Engineering, Shanghai Jiaotong University, Shanghai 200240, China; E-Mail: hudiepianpianlilei@hotmail.com; 2 School of Agriculture and Biology, Shanghai Jiaotong University, Shanghai 200240, China; 3 Center for Precision & Automated Agricultural Systems, Washington State University, 24106 North Bunn Rd., Prosser, WA 99350, USA; E-Mails: troy_peters@wsu.edu (T.P.); qinzhang@wsu.edu (Q.Z.); jj.zhang@wsu.edu (J.Z.)

**Keywords:** thermal imaging, energy balance, simulation model, fruit surface temperature, sunburn

## Abstract

The exposure of fruit surfaces to direct sunlight during the summer months can result in sunburn damage. Losses due to sunburn damage are a major economic problem when marketing fresh apples. The objective of this study was to develop and validate a model for simulating fruit surface temperature (FST) dynamics based on energy balance and measured weather data. A series of weather data (air temperature, humidity, solar radiation, and wind speed) was recorded for seven hours between 11:00–18:00 for two months at fifteen minute intervals. To validate the model, the FSTs of “Fuji” apples were monitored using an infrared camera in a natural orchard environment. The FST dynamics were measured using a series of thermal images. For the apples that were completely exposed to the sun, the RMSE of the model for estimating FST was less than 2.0 °C. A sensitivity analysis of the emissivity of the apple surface and the conductance of the fruit surface to water vapour showed that accurate estimations of the apple surface emissivity were important for the model. The validation results showed that the model was capable of accurately describing the thermal performances of apples under different solar radiation intensities. Thus, this model could be used to more accurately estimate the FST relative to estimates that only consider the air temperature. In addition, this model provides useful information for sunburn protection management.

## 1. Introduction

Sunburn on apple surfaces is a quality defect that results in culled apples. The exposure of apple surfaces to direct sunlight results in sunburn when the area becomes hot enough to cause cell tissue damage. Sunburn is a major economic problem in marketing fresh apples and can cause significant economic losses for some apple varieties, such as “Fuji”, “Jonagold”, and “Granny Smith”. Many orchardists apply overhead irrigation water to cool the fruit in an attempt to avoid sunburn. Temperature influences many biological processes in fruit development and affects the fruit size, colour, sugar content, acid content, nutritional quality, sunburn injury, and pest development [1–5]. Hence, temperature is an important parameter that affects fruit quality.

Methods for directly measuring the fruit surface temperature (FST) include pushing the bulb of a thermometer beneath the surface of the fruit, inserting thermocouples into the fruit, and placing thermocouples on the fruit surface. In addition, infrared thermometry and thermographic imaging are used for non-contact measurements.

Pushing the sensory bulb of a thermometer beneath the fruit's skin has commonly been used to monitor apple surface temperature [6–10]. However, this process wounds the fruit surface, affects the measurement and does not provide a direct measure of surface temperature. Therefore, this method should not be considered a decisive method for measuring fruit surface temperature [11]. Although the insertion of thermocouples into the fruit could make temperature measurements easier, this process wounds the fruit [12], and the measurements cannot be repeated on the same fruit [13,14] at the same location. To solve these problems, researchers have placed thermocouples on the fruit surface using small fabric adhesive bandages [15,16]. However, the adhesive bandages change the fruit surface conditions, including the reflective radiation properties, and result in less accurate temperature measurements.

Furthermore, the above measurements do not consider the spatial heterogeneity of the FST. The gradually changing position of the sun throughout the day causes the location of the highest temperature point on the fruit surface to change. Therefore, to measure the peak fruit surface temperature throughout the day, it is also important to change the measurement location on the fruit surface. Infrared thermometry and thermography can be used for non-destructive and remote determinations of FSTs because they detect the long-wave infrared radiation emitted from the fruit [17] and can be used to dynamically observe the highest temperature location on the fruit as it changes throughout the day. In addition, changes in the FST due to varying solar radiation can change the physiological and biochemical processes of the fruit due to active, natural, protective mechanisms (e.g., induction of HSPs genes). The associated changes in the form of heat balance on the fruit surface can be monitored instantly and remotely by using thermographic imaging [18]. Thus, infrared thermography shows great potential for detecting temperature differences on apple surfaces [2].

Although the temperature dynamics several fruits within a canopy could be monitored via infrared thermometry, thermography, or by an infrared camera, monitoring the temperature dynamics of all fruits throughout the entire growing season remains challenging. Modelling fruit surface temperatures promises to help overcome such difficulties. In addition, FST models could help guide orchardists when applying overhead cooling water. Thus, modelling fruit surface temperatures could save significant amounts of water and pumping energy.

Although modelling fruit surface temperature is meaningful and useful to orchardists, it is rarely performed. In some studies [19,20], air temperature has been considered rather than fruit temperature. However, theoretical and experimental evidence shows that the fruit temperature can be 10 °C higher than the air temperature under sunny conditions [9,10,21]. Evans [22] and Saudreau *et al.* [8] modelled the temperature dynamics of detached fruits based on climatic factors and used measured fruit temperature data from inserted thermocouples to verify their results. A linear regression of Evans's model with measured results for detached apples resulted in an R^2^ value of 0.73. The root mean square error between Saudreau's model estimation and the thermocouple measurements at a 1 min time step was approximately 0.8 °C.

In this study, a model for estimating the highest FST using measured weather data was developed. This model was validated by using data collected from thermal images. Because the solar radiation and maximum FST were highly correlated (r = 0.65) between 11:00 h and 17:00 h [16], this model considered the effects of sun exposure and leaf shading.

## 2. Materials and Methods

### 2.1. Model Description

Energy budgets in a biosphere have been used for various purposes [23], such as modelling animal body temperature [23], analysing human comfort [24], and designing greenhouses [25]. In addition, the temperature of growing fruit is governed by these energy budgets:

(1) $${\text{E}}_{Lh}+{\text{E}}_{Sh}+{\text{E}}_{Hs}+{\text{E}}_{Np}={\text{E}}_{Nr}$$

where E_Lh_ is the latent heat, E_Sh_ is the sensible heat, E_Hs_ is the heat storage, E_Np_ is the energy produced by net plant photosynthesis and E_Nr_ is the net radiation.

The heat energy balance of the fruit surface during growth is modelled by specifying the normal heat flux at any point on the fruit surface:

(2) $$\{\begin{array}{c} E_{\text{input}}=R_{\text{abs}}\\ E_{\text{output}}=\lambda_{E}+H+R_{e}+E_{f} \end{array}$$

where E_input_ is the input energy, E_output_ is the output energy, R_abs_ is the total incoming radiation (W/m^2^), R_e_ is the emitted thermal radiation by the fruit (W/m^2^), *λ_E_* is the loss of energy by evaporation (latent heat loss), and *H* is the loss or gain of sensible energy by convection. Furthermore, *E_f_* is the total heat transfer within the fruit, including that from the plant to the fruit, the fruit temperature gradient, and the metabolism activity. The flux of water from the plant to the fruit may induce heat fluxes (Dufour effect) or may occur because of a temperature gradient (Soret effect). However, the Soret and Dufour effects act at large time scales or in response to high temperature gradients, such as those in drying wood. Regarding the heat flux at the fruit surface, these two heat fluxes are usually small compared with conduction and radiation. Compared with the exchange between the fruit surface and the surrounding environment, the heat release from metabolic activity within the fruit and the energy exchange between the plant and the fruit are small. In addition, *E_f_* includes the heat fluxes (H_c_) that are produced during heat transfer by conduction (Fourier effect) and can be calculated by using Equation (A.1) in Appendix A. The highest FST represents only one point temperature on the fruit surface at some time, and the apple thermal conductivity is approximately 0.5 Wm^−1^K^−1^. The heat fluxes (H_c_) within the fruit are not considered relative to other fluxes. Thus, *E_f_* is assumed to be small enough to be neglected at any particular point in time, and the input energy is approximately equal to the output energy. Therefore, the heat energy balance equation of the fruit surface could be simplified as follows:

(3) $$R_{\text{abs}}=\lambda_{E}+H+R_{e}$$

The difference between R_abs_ and R_e_ is the net absorbed radiation (R_n_) (W/m^2^). The FST is influenced by many environmental factors, such as solar radiation, air temperature, wind, and humidity. These factors are described below.

#### 2.1.1. Radiation

**Figure 1.**
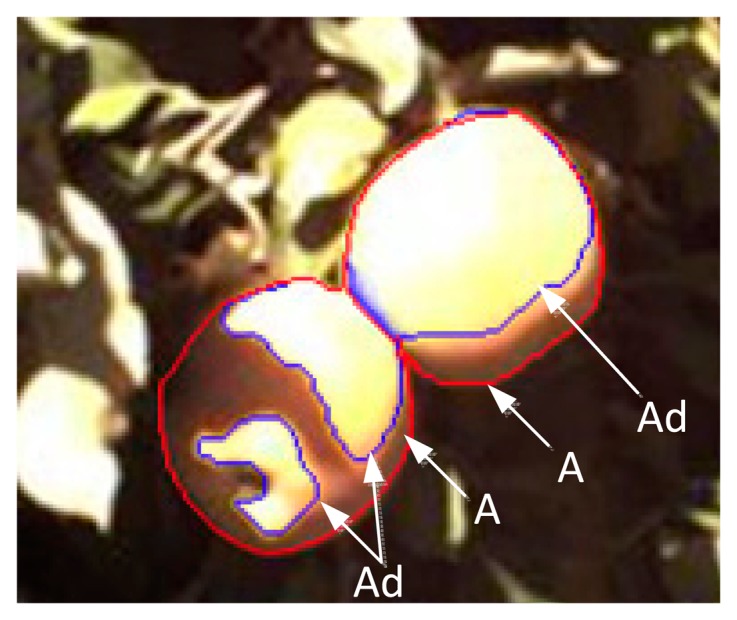
Illumination area estimation. A is the estimation of the maximum projected sunlit fruit surface area in the incident sunlit direction; A_d_ is the estimation of the projected sunlit fruit surface area in the direction of the incident sunlight.

The total incoming radiation R_abs_ includes the incoming net short-wave radiation (R_ns_) and the total incoming net long-wave radiation (R_nl_), as defined in the following set of equations:

(4) $$\{\begin{array}{c} R_{\text{abs}}=R_{ns}+R_{nl}\\ R_{ns}=\frac{A_{d}}{A}\left(1-\alpha \right)R_{s}\\ R_{nl}=\varepsilon_{a}\sigma {\left(T_{a}+273\right)}^{4}+\frac{A_{r}}{A^{'}}\varepsilon_{g}\sigma {\left(T_{\text{gmean}}+273\right)}^{4} \end{array}$$

where R_ns_ is the incoming short-wave radiation (Wm^−2^), *α* is the reflectance of the apple surface (approximately 0.6 [22]), R_nl_ is the incoming long-wave radiation (Wm^−2^), σ is the Stephan–Boltzmann constant (5.67E^−8^ Wm^−2^T^−4^, with T in Kelvin), T_a_ is the surrounding air temperature (°C), T_gmean_ is the mean temperature (°C) of the surrounding ground and leaves, A is the estimated maximum projected sunlight on the fruit surface area in the incident sunlit direction, A_r_ is the projected area exposed to reflected radiation from the ground and canopy, and A' is the total surface area of the fruit (e.g., 4πr^2^ for a sphere). Furthermore, A_d_ is an estimate of the projected sunlit fruit surface area in the direction of the incident sunlight. Because this study is used for preventing sunburn, we were primarily interested in estimating the maximum temperature of the fruit surface (*i.e.*, relatively small areas where sunburn occurs). In this study, the A_d_/A values were estimated from RGB exposed images (as illustrated in Figure 1). The lighting scenario on the fruit surface could be the surface that is perpendicular to the light direction or is partially or completely shaded by an object [26]. To estimate the maximum temperature of the fruit surfaces under different illumination, the A_d_/A ratios were set at 1, 0.8, 0.6, 0.5, 0.4, 0.2, and 0.0. A ratio of 0.0 represents a fruit that is completely hidden by leaves with no exposure to sunlight. The effects of different A_d_/A ratios on FST were analysed. In addition, *ε*_g_ is the emissivity of the ground. The A_r_/A' ratio (*i.e.*, exposed to reflected radiation from the ground and canopy) is an empirical parameter (maximum is 0.6). In addition, *ε*_a_ is the emissivity of the atmosphere and is given by Equations (A.2) and (A.3) in the Appendix.

The emitted radiation (R_e_) is calculated as follows:

(5) $${\text{R}}_{e}=\varepsilon_{\text{f}}\sigma {\left({\text{T}}_{\text{fs}}+273\right)}^{4}$$

where ε_f_ is the emissivity of the fruit, 0.95 [8], and T_fs_ is the temperature (°C) at the fruit surface.

#### 2.1.2. Convection

The sensible heat gained or lost by convection (*H*) is expressed as follows [22]:

(6) $$H=c_{p}g_{a}\left(T_{fs}-T_{a}\right)$$

where *c_p_* is the specific heat capacity of the air at 29.3 J mol^−1^°C^−1^. In addition, g_a_ is the boundary layer conductance of the turbulent heat flow transfer from the fruit surface to the air and can be estimated as

(7) $$g_{a}=1.4\times 0.135\times \sqrt{\frac{u}{d}}$$

where *d* is a characteristic length estimated by multiplying the fruit diameter by 0.84, which is an empirical parameter when the apple is treated as a sphere [22]. Furthermore, *u* is the wind speed. The constant 1.4 was empirically determined for application in outdoor environments that were influenced by air temperature and humidity [23].

#### 2.1.3. Transpiration

The latent heat flux (*λ_E_*) due to transpiration is expressed as follows [23]:

(8) $$\begin{array}{c} \lambda_{E}=\lambda g_{w}\rho_{\text{air}}\frac{e_{s}\left(T_{fs}\right)-e_{a}}{P}\lambda g_{w}\rho_{\text{air}}\frac{e_{s}\left(T_{fs}\right)-e_{s}\left(T_{a}\right)}{P}+\lambda g_{w}\rho_{\text{air}}\frac{e_{s}\left(T_{a}\right)-e_{a}}{P}\\ ≅\lambda g_{w}\rho_{\text{air}}\frac{\Delta \left(T_{fs}-T_{a}\right)}{P}+\lambda g_{w}\rho_{\text{air}}\frac{e_{s}\left(T_{a}\right)-e_{a}}{P} \end{array}$$

where *e_s_*(*T_fs_*) is the vapour pressure at the FST, *T_fs_* (°C); *e_s_*(*T_a_*) is the vapour pressure at the air temperature *T_a_* (°C); *λ* is the latent heat of vaporisation (2.429 MJ/Kg); and Δ is the increasing rate of the saturation vapour pressure at the dew point temperature (kPa°C^−1^). Furthermore, *g_w_* is the fruit surface conductance to water vapour and *ρ_air_* is the air density (Kg m^−3^). The equations used to calculate the *e_s_*(*T_a_*), Δ and *ρ_air_* are provided in Appendix A.

### 2.2. Model Implementation

The general objective of the model is to simulate the dynamics of the highest fruit surface temperatures *T_fs_* based on the corresponding variations in the air characteristics and incoming radiation (solar radiation and long-wave radiation). The *T_fs_* can be estimated by substituting (Equations (4)–(8)) by (Equation (3)) and combining (Equations (A.2)–(A.9)) to obtain the following a quartic equation:

(9) $${\text{T}}_{\text{fs}}+273=-\frac{\varepsilon_{\text{f}}\sigma {\left({\text{T}}_{\text{fs}}+273\right)}^{4}}{c_{p}g_{a}\frac{\lambda g_{w}\rho_{\text{air}}\Delta }{P}}+\frac{R_{\text{abs}}+c_{p}g_{a}T_{a}+\frac{\lambda g_{w}\rho_{\text{air}}\Delta T_{a}}{P}-\frac{\lambda g_{w}\rho_{\text{air}}\left(e_{s}\left(T_{a}\right)-e_{a}\right)}{P}}{g_{a}c_{p}\frac{\lambda g_{w}\rho_{\text{air}}\Delta }{P}}+273$$

The optical and thermal properties were provided in Section 2.4. The meteorological data for the model are detailed in Section 2.5. The apple characteristics that were required for the model include an estimation of the maximum projected sunlit fruit surface area in the incident sunlit direction and an estimation of the projected sunlit fruit surface area in the direction of the incident sunlight.

### 2.3. Field Measurements

#### 2.3.1. Fruit Material

**Figure 2.**
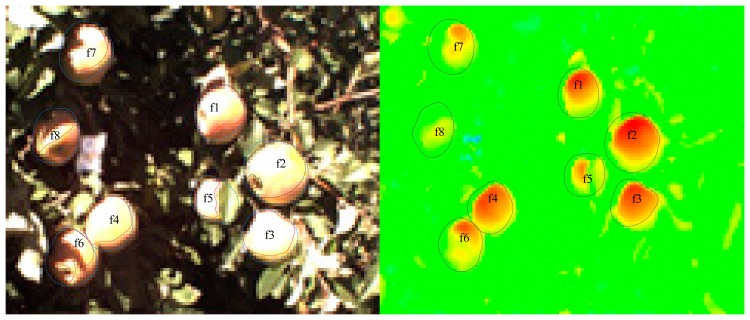
RGB image (**left**) and thermal image (**right**) in the field. In the RGB image, the red region is an estimation of the projected sunlit fruit surface area in the direction of the incident sunlight, (A_d_) and the blue region is an estimation of the maximum projected sunlit fruit surface area in the incident sunlit direction (A). In the thermal image, the black region is the fruit region.

Field data collection was conducted in August and September, 2013, at Washington State University at the Roza Research Farm near Prosser, WA (46.29 N, 119.73 W). Tree rows were arranged in a north-south direction. A set of 41 “Fuji” apples at heights of 1.5 m to 2.5 m on ten trees were randomly selected in the orchard. Thermal images of each apple (an example is shown in Figure 2) were captured using a FLIR A615 infrared camera (FLIR Systems Inc., Portland, OR, USA) to detect the temperature of the fruit surface. These images were calibrated using a BB701 Blackbody Calibrator (OMEGA Engineering, Inc., Stamford, CT, USA).

Apple sunburn normally occurs between 11:00 h and 17:00 h. To acquire information regarding sunburn development, fruit images were acquired from 11:00 h to 18:00 h (midday at 13:00 h) at 15 min intervals in August and from 11:00 h to 17:00 h (midday at 12:00 h) in September to reflect the earlier sunset. The distance of the camera's lens to the monitored apple surfaces was readjusted manually to approximately 0.7 m, and the camera was automatically refocused before each image acquisition. To obtain the highest FST value, the shooting angles were manually adjusted every hour following direct sunlight to maintain consistent angles for the images with the incident sunlight while avoiding casting a shadow over the viewing area. The highest fruit surface temperature measured by the thermal images was more precise than the highest fruit surface temperatures measured by the temperature sensors, including the thermistors and thermocouples. Thermal images could record a large number of temperature points at the same time, ideally allowing users to obtain the maximum temperature on the fruit surface.

#### 2.3.2. Measurement of Highest FST

The RGB images were acquired using a digital colour camera (GC1290, Allied Vision Technologies Inc., Newburyport, MA, USA) from the same location to record the illumination conditions of the monitored apples. The illumination levels were estimated by the ratio between the projected sunlit fruit surface area in the direction of the incident sunlight (A_d_) and the maximum projected sunlit fruit surface area in the incident sunlit direction (A). The A_d_/A ratio equalled 1, while the ratio was 0.9 to 1, and so on. Based on the exposure image of RGB, the selected exposure region was used to estimate A_d_, and the contour region was used to estimate A. Our experiment was conducted at the Roza Research Farm at Washington State University near Prosser, WA (46.29 N, 119.73 W) under mainly sunny conditions. Because sunburn occurs on sunny days, we used the exposure image of RGB to estimate the A_d_.

By using thermal image processing software (FLIR EXAMINIR; FLIR Systems Inc.), the fruit surface on the thermal image was manually obtained and the surface temperature (including the mean temperature, maximum temperature and minimum temperature) was obtained from those thermal images. Improved surface temperature measurement accuracy also requires inputs from the surrounding environment, including parameters like distance, atmospheric temperature, and relative humidity. For example, based on the thermal image in Figure 2 (right), measuring the highest FST step includes the following five steps: (1) open the thermal image in the thermal processing software (FLIR EXAMINIR); (2) input the surrounding environmental parameters, including the distance between the fruit and the thermal camera, atmospheric temperature, relative humidity and fruit emissivity; (3) select the region of each fruit, as shown in Figure 2 (right); (4) calculate the highest FST of each fruit region; (5) calculate the highest average FST of the fruits at the same level according to the illumination levels classified by the illumination area based on the image. For example, f1, f2, f3 and f4 are at the same level, and the highest FST at a given level and at a given time is the average value of the highest FST.

### 2.4. Optical and Thermal Properties

Two parameters were used in the model that were related to radiation at the fruit surfaces and must be known prior to simulation. These parameters include the emissivity (ε_f_) for long-wave IR radiation (Equation (4)) and the albedo or reflectance (α) for short-wave radiation (Equation (4)). For apples, ε_f_ is generally accepted as 0.94–0.97 [27]. To investigate the influences of these values on the model, the minimum and maximum values of the fruit emissivity (ε_f_) (0.90 and 1.00, respectively) corresponded to relative variations of ±5% when compared with an ε_f_ of 0.95. The α value is approximately 0.6. However, this value varies with the incidence angle of sunlight, and according to the literature [8,10], its relative variation is approximately ±20% of 0.6.

Another important property for the model is the surface conductance to water vapour diffusion g_w_. It is difficult to measure g_w_, and measured g_w_ values are often inaccurate. In this case, a g_w_ value of 5 × 10^−5^ ms^−1^ was used [28,29]. The surroundings have a large influence on g_w_, which varied from 0 (no transpiration process) to +200%. To investigate the influences of these parameters on the model predictions, a sensitivity analysis of these parameters was conducted in Section 3.2.2.

### 2.5. Measurements of the Climatic Variables

An automatic weather station [30] located approximately 400 m upwind from the testing orchard provided meteorological data, including the air temperature, relative humidity, wind speed, wind direction and soil temperature at 2 m above the ground. The short-wave radiation R_s_, from which the direct radiation was estimated, was recorded using a CS300-L Pyranometer (CS300, Campbell Scientific, Inc. Logan, UT, USA) every 10 s, and the averaged values of the 15 min readings (90 data points) were used as the measured data.

## 3. Results and Discussion

### 3.1. Difference Comparisons of Air Temperature and FST

**Figure 3.**
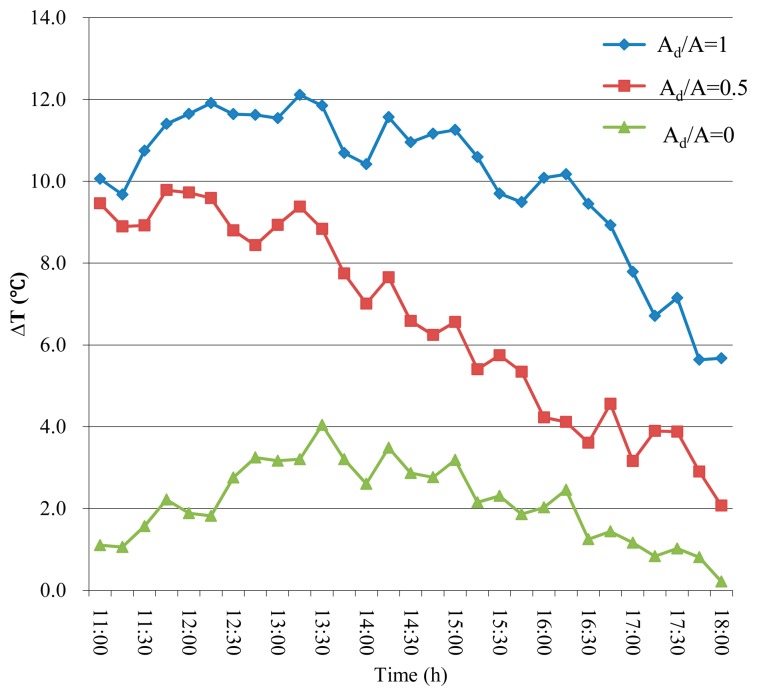
The mean difference between the hottest FST and the air temperature from 11:00 h to 18:00 h for three illumination levers (A_d_/A = 0, 0.5, 1).

Evidence suggests that fruits can be heated to temperatures that are significantly higher than air temperatures in the field. Figure 3 presents the difference between the air temperature and the highest fruit surface temperature under different area ratios of illuminated surface at different times of the day. According to Figure 3, this difference varied from 0 to 12.0 °C during the 11:00 h to 18:00 h time window. The largest differences reached 12.0 °C around solar noon for the fruit with an A_d_/A ratio near 1.0, 9.8 °C for the fruits with an A_d_/A ratio of 0.5, and 3.2 °C for the fruits with an A_d_/A ratio of 0. These results suggest that different proportions of illumination largely influence the FST. The maximum differences occurred at approximately 14:00 h (*i.e.*, one hour after solar noon), mainly because the solar radiation was highest between midday and two hours after midday, which would cause the highest FST. However, the validation measurement indicated the FST reached its peak value if the wind speed was not very high. In almost all of our field measurements, we did not observed any cases where high wind affected the peak FST value. The FST peaked while the air temperature did not significantly increase. Next, the temperature differences gradually decreased. For shaded fruit (A_d_/A = 0), the total difference between air temperature and fruit surface temperature was not notable, and the fruit surface temperature was estimated using the air temperature.

### 3.2. Model Evaluation

#### 3.2.1. Model Validity

**Figure 4.**
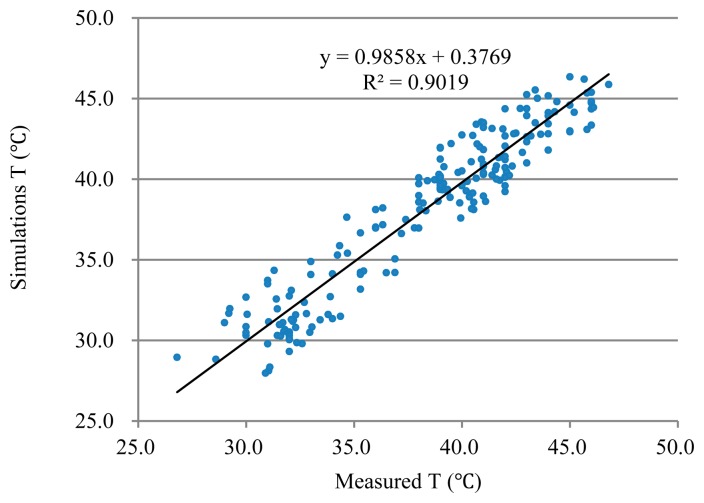
The linear regression of the simulated temperature for nine days.

**Figure 5.**
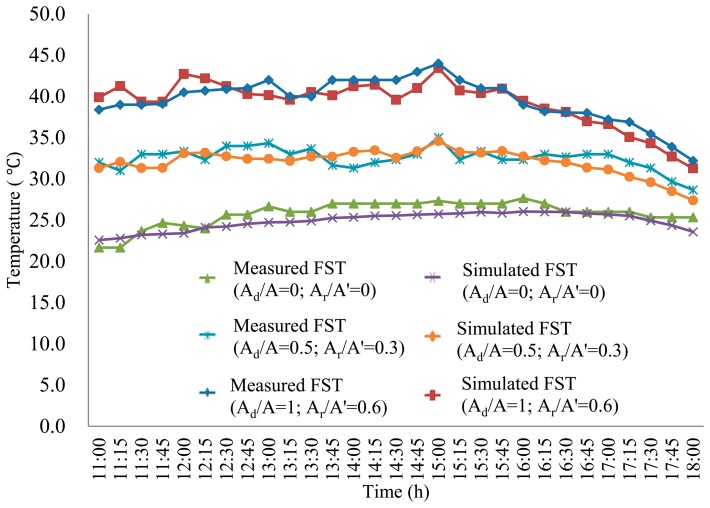
Temperature *vs.* time: Averaged simulation results and measurements for the various apples at different illumination levels on DOY 232.

The highest fruit surface temperatures with an A_d_/A value of 1 from 200 different thermal images collected on nine different days (Days-of-Year [DOY] 215, 224, 232, 239, 242, 252, 256, 262, and 263) were used to validate the model. The model inputs consisted of the fruit property parameters and the measured microclimate parameters, including air temperature, humidity, wind speed, and solar radiation. A simulation time step of 15 min was used. The simulated FST values from the developed model were compared with the 200 measured data points. The linear regression of the simulated FST and measured FST had a coefficient of determination (R^2^) of 0.90, with an absolute error of less than 2.0 °C. The error distribution appeared to be normally distributed (mean of 0.013 and standard deviation of 1.7), as shown in Figure 4. The RMSE was less than 2.0 °C (Figures 4 and 5). Compared with previous analytical studies (R^2^ of 0.73) [8,22], the presented study estimated peak FST more accurately. The use of thermal images more precisely measured the effects of a realistic environment (*i.e.*, unsteady and non-homogeneous heat fluxes) on the peak temperature dynamics of the fruit surface.

To highlight the temperature dynamics of the fruit surface, the highest FSTs under A_d_/A values of 1, 0.5 and 0 on DOY 232 are shown in Figure 5. The largest standard deviation was 1.9 °C within the allowable error of 2.0 °C, which validated the overall accuracy of the developed model for simulating FST. Statistically, we observed that the simulated FST was more precise when the A_d_/A value was 1 for an A_r_/A' value of 0.6, when the A_d_/A value was 0.5 for an A_r_/A value of 0.3, and when the A_d_/A value was 0 for an A_r_/A value of 0.6.

#### 3.2.2. Sensitivity Analysis

**Figure 6.**
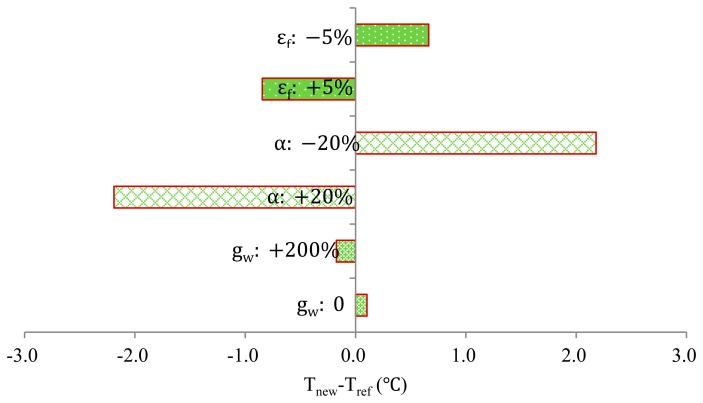
Sensitivity analysis of the model to fruit physical parameters (the surface conductance to water vapour diffusion g_w_, the fruit surface reflectance α and the emissivity (ε_f_) of the fruit surface).

Figure 6 presents the results of a sensitivity analysis of the model with selected fruit physical parameters. A negative value indicates a decrease in temperature for a corresponding decrease in the input parameter or *vice versa.* The reference fruit surface temperature (T_ref_) of the output value of the model is shown. While some parameters were changed, other parameters were held constant at reference values of α = 0.6, g_w_ = 5 × 10^−5^ ms^−1^, and ε_f_ = 0.95. The changes of α (α: ±20 %) resulted in larger changes in FST (*i.e.*, approximately 2.0 °C). Changes in physical parameters, such as the emissivity of the fruit (ε_f_: ±5 %) and the surface conductance to water vapour diffusion (g_w_:0%–+200%), only resulted in minor variations in FST (less than 0.5 °C). When the emissivity (ε_f_) was increased by 5%, the fruit emitted more energy, which decreased the FST. When the fruit surface reflectance *α* was reduced by 20%, the amount of energy absorbed by the fruit increased, which caused the FST to increase. Additionally, when the surface conductance to water vapour diffusion g_w_ increased by 200%, the evaporative cooling increased and caused a decrease in FST. This situation was reversed when g_w_ was set to 0.

**Figure 7.**
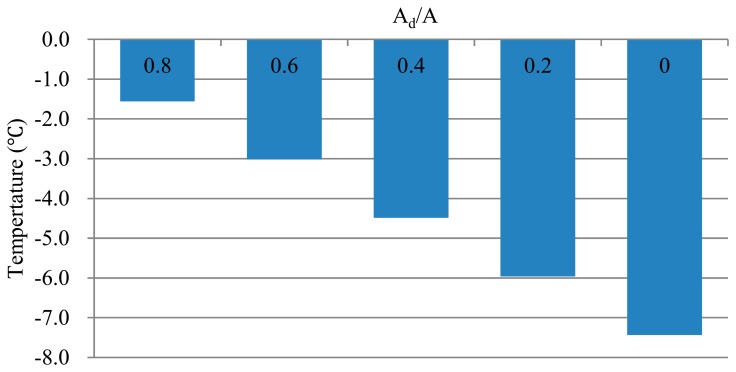
Sensitivity analysis of the model to different illumination area.

As expected, the energy received by the apples from direct sunlight is a major factor that affects FST [31]. As shown in Figure 7, different illumination areas largely influenced the peak FST. The reference temperature corresponded to the FST when the A_d_/A ratio was equal to 1. When the A_d_/A ratio was equal to 0.6 or 0.4, the temperature difference was 1.5 °C. The difference between all of the shaded areas (*i.e.*, A_d_/A = 0) and all of the sunny areas (*i.e.*, A_d_/A = 1) reached 7.4 °C.

#### 3.2.3. Impact Analysis of Fruit Temperature and Thermal Parameters on FST

The model can be used to analyse the relevance of different thermal parameters (the surface conductance to water vapour diffusion g_w_, and the emissivity (ε_f_) of fruit surface) for predicting fruit temperature by using a sensitivity analysis. This point is critical because it shows which parameter is relevant when making decisions regarding conducting potential experiments. The sensitivity analysis (Figure 6) showed that only the parameters involved in the heat fluxes at the fruit surface were required to correctly predict the temperature on the fruit surface.

The values of g_w_ obtained from previous studies [28] were not well characterised because the relative variations could reach +200%. The three following factors could explain this large variation: (1) the difficulty in measuring g_w_ because its order of magnitude was 0.001 ms^−1^; (2) the deduction of this parameter from the VPD and other parameters according to (Equation (8)) (thus accurate measurements were not straightforward [29]); and (3) the g_w_ value varied with the fruit growth conditions and with fruit development [28]. As expected, increasing g_w_ (+200% of the original value) enhanced the transpiration process and cooled the entire fruit. This scenario decreased the FST by 0.2 °C. Although increasing g_w_ by +200% resulted in the apparent surface conductance to water vapour diffusion, no substantial decrease in the FST was observed (Figure 6). This finding suggested that the evaporation process could be neglected.

The sensitivity analysis revealed that the fruit emissivity ε_f_ was also an important parameter in the FST. Increasing ε_f_ (+5%) resulted in a decrease (−0.8 °C) in the FST. In contrast with the g_w_ value, the ε_f_ value was accurately estimated in previous studies [32]. The value of ε_f_ increased from 0.9 to 1.0, which resulted in a 1.5 °C decrease in FST. Thus, fruit emissivity had a relatively substantial effect on the FST compared with the g_w_.

The sensitivity of the model to thermal parameters (g_w_ and ε_f_ ) was relatively low. This phenomena was useful for investigating the effects of microclimates on fruit responses in terms of physiological disorders. Moreover, the FST is not sensitive to thermal parameters (g_w_ and ε_f_). Thus, the amount of energy received or released, as well as the time and length scale ratios between the heat fluxes at the fruit surface, were very important to the FST.

#### 3.2.4. Relationship between Microclimate and FST

**Figure 8.**
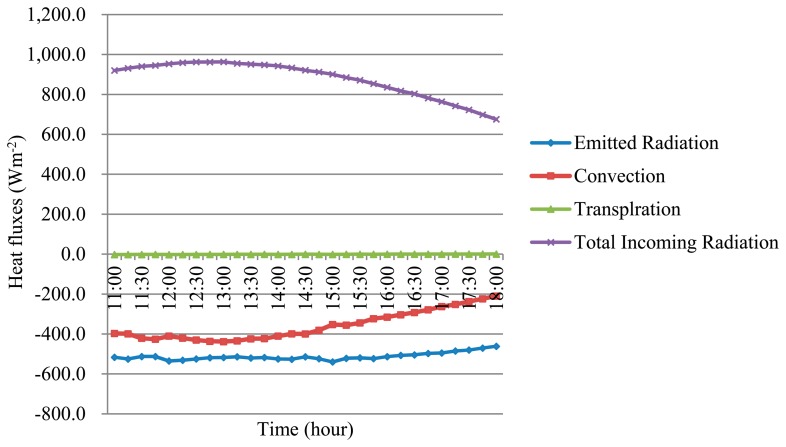
Heat fluxes at the surface of the apple: total incoming radiation (R_abs_), convection (H), transpiration (λ_E_), and emitted radiation by the apple surface (R_e_) on DOY 232.

To investigate the relationships between the environment and the FSTs, all of the heat fluxes at the fruit surface were calculated based on the simulated temperature of the model and by using (Equations (4), (6) and (8)). Figure 8 shows the amplitudes of the heat fluxes, including the total incoming radiation (R_abs_), convection (H), transpiration (λ_E_), and emitted radiation of the apple surface (R_e_) on DOY 232. A positive value of heat flux corresponds to a gain of heat (*i.e.*, the surface becomes warmer). A negative value of the heat flux corresponds to a loss of heat (*i.e.*, the surface becomes cooler).

The loss of energy caused by evaporation (*i.e.*, latent heat loss) was negligible and smaller than the loss caused by the total incoming radiation and convection processes. Between 11:00 h and 18:00 h, the changes in the latent heat loss and emitted radiation were relatively flat. A similar trend was observed regarding changes in the heat fluxes on the fruit surface for the other days observed.

In addition to estimating the FST dynamics, the model simulated the heat flux dynamics on the fruit surface. Radiation and convection govern the dynamics of fruit surface temperature, and should be accurately implemented. The results validated the methods of estimating FST, as shown by (Equations (4), (8) and (14)).

The forced convection used in the model (Equation (8)) was caused by the difference between the FST and air temperature. Radiation and convection remained the driving processes that governed the FST and were substantially affected by the distribution of tree foliage. Therefore, the characterisation or modelling of the air within a tree canopy is critical for accurate FST modelling.

In addition, the maximum FST was only simulated on clear, sunny days because fruit sunburn occurs under these conditions. In future research, the model will need to be modified to estimate the FST under different weather conditions (such as cloudy, overcast, or rainy conditions). In addition, the influences of different fruit varieties, wind directions, canopy shape and density should be considered. The model can be used to prevent sunburn on other fruits as well.

## 4. Conclusions

A physical model simulating the variations of the maximum fruit surface temperature was developed in this study. The model was based on an energy balance and field data. Model outputs were compared with fruit surface temperatures that were measured from thermal images. The linear regression of the simulated FST and the measured FST had a coefficient of determination (R^2^) of 0.90 and a mean absolute error of less than 2.0 °C. The obtained results showed that the model accurately described the thermal performance of the apples and was more accurate than the estimates that are usually made based on air temperature. This finding suggested that the model could be used to study microclimatic effects on fruits, such as sunburn, and to provide useful information for sunburn protection management. Next, this model should be used to define temperature and time thresholds to automate fruit evaporative cooling systems and avoid sunburn.

## Appendix A. Extended Fruit Energy Balance

The heat flux (H_c_) was produced by heat transfer through conduction (Fourier effect) within the fruit and under conditions that only considered the incident sunlit direction. This flux can be modelled by Fourier's Law as follows:

(A.1) $${\text{H}}_{\text{c}}=\text{k}\frac{\text{dT}}{\text{dt}}\text{S}$$

where k is the thermal conductivity (Wm^−1^K^−1^), $\frac{\text{dT}}{\text{dt}}$ is the temperature gradient (°C) and S is the surface are (m^−2^) at the incident sunlit direction on the fruit surface. The emissivity of the atmosphere *ε*_a_ is given by the following equation [33]:

(A.2) $$\varepsilon_{a}=1.72{\left(\frac{e_{a}}{T_{a}}\right)}^{1/7}$$

where *e*_a_ is the actual vapour pressure (kPa). The actual vapour pressure is the saturation vapour pressure at the dew point temperature (T_dew_) (°C) and is expressed as follows:

(A.3) $$e_{a}={\text{e}}_{{\text{T}}_{\text{dew}}}^{\text{S}}=0.6108\exp\left(\frac{17.27T_{\text{dew}}}{T_{\text{dew}}+237.3}\right)$$

The air density *ρ_air_* can be determined using the following equation [34]:

(A.4) $$\rho_{\text{air}}=\frac{1000P}{T_{Kv}R}=3.486\frac{P}{T_{Kv}}$$

where *R* is the specific gas constant of 287 (J Kg^−1^ K^−1^) and *T_Kv_* is the mean virtual temperature (K):

(A.5) $$T_{Kv}=1.01\left(T_{\text{mean}}+273\right)$$

Here, T_mean_ is the mean daily temperature for 24 h calculation time steps.

(A.6) $$P=P_{o}{\left(\frac{T_{Ko}-\alpha_{1}(z-z_{0})}{T_{Ko}}\right)}^{\frac{g}{\alpha_{1}R}}$$

where *P* is the atmospheric pressure at elevation Z (kPa), P_0_ is the atmospheric pressure at sea level (101.3 kPa), Z is the elevation (m), Z_0_ is the elevation at the reference level (m), *g* is the gravitational acceleration (9.807 m s^−2^), *R* is the specific gas constant (287 J kg^−1^ K^−1^), α_1_ is the constant lapse rate of moist air (0.0065 K m^−1^), and T_k0_ is the reference temperature (k) at elevation Z_0_. The elevation Z_0_ is generally considered as sea level for standardised calculations.

The American Society of Civil Engineers (ASCE) assumed the standard atmosphere to be P_0_ = 101.3 kPa at Z_0_ = 0 and T_k0_ = 293 K for T_mean_ = 20. Therefore,(Equation (A.5)) simplifies to

(A.7) $$\text{P}=101.3{\left(\frac{293-0.0065\text{z}}{293}\right)}^{5.26}$$

where Δ is the increasing rate of the saturation vapour pressure at the dew point temperature (kPa°C^−1^).

(A.8) $$\Delta =\frac{2503\exp(\frac{17.27T_{a}}{T_{a}+237.3})}{T_{a}+237.3}$$

where, *e_s_*(*T_a_*) is the vapour pressure at the air temperature Ta (°C)

(A.9) $$e_{s}\left(T_{a}\right)=0.6108\exp\left(\frac{17.27T_{a}}{T_{a}+237.3}\right)$$
